# Reliability and Validity of a 20-s Alternative to the Wingate Anaerobic Test in Team Sport Male Athletes

**DOI:** 10.1371/journal.pone.0114444

**Published:** 2014-12-04

**Authors:** Ahmed Attia, Younes Hachana, Helmi Chaabène, Abdelmajid Gaddour, Zied Neji, Roy J. Shephard, Mohamed Souhaiel Chelly

**Affiliations:** 1 Research Unit « Sport Performance & Health», High Institute of Sport and Physical Education of Ksar Saîd, University of “Manouba”, Tunis, Tunisia; 2 High Institute of Sport and Physical Education of Ksar Said, University of “Manouba”, Tunis, Tunisia; 3 Faculty of Kinesiology and Physical Education, University of Toronto, Toronto, Ontario, Canada; Glasgow University, United Kingdom

## Abstract

The intent of this study was to evaluate relative and absolute reliability of the 20-s anaerobic test (WAnT_20_) versus the WAnT_30_ and to verify how far the various indices of the 30-s Wingate anaerobic test (WAnT_30_) could be predicted from the WAnT_20_ data in male athletes. The participants were Exercise Science majors (age: 21.5±1.6 yrs, stature: 0.183±0.08 m, body mass: 81.2±10.9 kg) who participated regularly in team sports. In Phase I, 41 participants performed duplicate WAnT_20_ and WAnT_30_ tests to assess reliability. In Phase II, 31 participants performed one trial each of the WAnT_20_ and WAnT_30_ to determine the ability of the WAnT_20_ to predict components of the WAnT_30_. In Phase III, 31 participants were used to cross-validate the prediction equations developed in Phase II. Respective intra-class correlation coefficients (ICC) for peak power output (PPO) (ICC = 0.98 and 0.95) and mean power output (MPO) (ICC 0.98 and 0.90) did not differ significantly between WAnT_20_ and WAnT_30_. ICCs for minimal power output (PO_min_) and fatigue index (FI) were poor for both tests (range 0.53 to 0.76). Standard errors of the means (SEM) for PPO and MPO were less than their smallest worthwhile changes (SWC) in both tests; however, PO_min_ and FI values were “marginal,” with SEM values greater than their respective SWCs for both tests values. Stepwise regression analysis showed that MPO had the highest coefficient of predictability (R = 0.97), with PO_min_ and FI considerable lower (R = 0.71 and 0.41 respectively). Cross-validation showed insignificant bias with limits of agreement of 0.99±1.04, 6.5±92.7 W, and 1.6±9.8% between measured and predicted MPO, PO_min_, and FI, respectively. WAnT_20_ offers a reliable and valid test of leg anaerobic power in male athletes and could replace the classic WAnT_30_.

## Introduction

Classical analyses of human physical performance suggested three primary energy sources: an anaerobic power (largely phosphagen based, and typically depleted within 2–4 s), an anaerobic capacity (limited largely by lactate accumulation, and exhausted within about 45 s) and an aerobic power capable of sustaining effort for much longer periods [1], [2]. The 5 s and 30 s power output measurements of the standard 30-s Wingate Anaerobic Test (WAnT_30_) were designed to examine the first two of these energy reserves [3]. The WAnT_30_ is both a reliable and a valid test [4]–[6], and is the most popular method of evaluating anaerobic ability. Although there are other potential measures such as short sprints [7], continuous vertical jumping [8] and elliptical all-out tests [9]. However, During the 30 s maximal effort of the WAnT_30_, the accumulation of [H^+^] as a by product of anaerobic glycolysis results in a drop in blood pH [10]. The increased acidity impairs activity of the enzymes involved in energy release and reduces maximal muscle fibre recruitment [11]. Further, the acute increase of blood glucose usage can result in a temporary hypoglycemia [12]. Undesirable side effects of the 30 s test thus include headache, vomiting, dizziness, and nausea [13]. In athletic applications where frequent assessments are required, awareness of these side effects may lead to less than maximal efforts during repeated testing, with a negative impact upon the reliability and validity of the test [14]. A previous study has shown that a 10 s reduction of test duration reduces physical discomfort in more than 90% of participants [14], leading to the view that a shortening of the test protocol might be helpful in some athletic and clinical applications.

The aerobic contribution is a further argument in favour of shortening the test. Although the objective of the WAnT_30_ is to assess peak anaerobic power and anaerobic capacity, it is recognized that over the 30 s of maximal effort, some ATP regeneration occurs through oxidative phosphorylation [15]. The magnitude of this aerobic contribution has been variously estimated at 9–19% [16], 28% [3]? 40% [17] or even 44% [18], although [19] found that the aerobic contribution was only 16% even during the final 5 s of the test.

Previous study [14] have found no difference of peak power output (PPO) between 20 s and 30 s tests, since PPO is usually attained in the first 5 s of effort. Furthermore, it appears that the mean power output (MPO), the minimum power output (PO_min_), and the fatigue index (FI) as determined by a WAnT_30_ can be predicted accurately from a WAnT_20_ [14]. The studies cited all emphasized the decrease in physical discomfort when using the WAnT_20_, but observations were limited to non-athletes, and the reliability [1] of the WAnT_20_ relative to the WAnT_30_ was not formally established. Moreover, the prediction algorithms developed in these earlier reports were not validated by subsequent, independent studies.

Therefore, the objectives of the present study were to evaluate the reliability of the WAnT_20_ relative to the WAnT_30_, and to verify how far the data obtained from the WAnT_30_ could be used to predict the traditional WAnT_20_ measures in male team-sport athletes.

## Methods

### Participants

The human subject committee of the local university (i.e., High Institute of Sport and Physical Education of Ksar Said, Tunis, Tunisia) approved the study in accordance with the 1975 Declaration of Helsinki. Eighty-one competitive, male team-sport athletes (age: 21.5±1.6 yrs, stature: 0.183±0.08 cm, body mass: 81.2±10.9 kg) were recruited. All were Exercise Science and Physical Education students, participating in regular training and competitive sports team schedules (soccer, basketball, rugby, and handball); they had an average of 6.3±0.9 yrs of training). All participants were fully informed of the nature of the study and they provided written and informed consent in accordance with accepted policy statements regarding the use of human participants.

### Procedures

The standard 30-s Wingate Anaerobic Test and WAnT_20_ tests were both performed on a friction belt cycle ergometer (Monark 894 E Peak Bike, Weight Ergometer, Vansbro, Sweden) with a basket weight loading system interfaced to a microcomputer. Monark Anaerobic Test; Software version 2.22 was used to record second-by-second power output throughout the test. The external loading was set at 7.5% of the individual's body mass [4], [14]. Participants were familiarized and habituated to the test protocol on two separate occasions prior to collection of definitive data; they performed high-velocity sprint exercises interspersed with 3 min of rest, so as to minimize continued test learning during the definitive experiment. Optimal saddle and handlebar positioning were determined for each subject prior to their first test, and the same placements were used in subsequent tests. Toe clips were used throughout.

Definitive tests were preceded by a standardized warm-up [20] that comprised three 30-s periods of active rest (zero-resistance pedalling at 60 rpm) alternating with three 30-s bouts of exercise at increasing external resistance (25, 50, and 75% of the test resistance, respectively). Participants were instructed to pedal at maximal effort throughout the definitive test. Both WAnT_30_ and WAnT_20_ tests began from a standstill position, with full application of the predetermined resistance. Participants were allowed to stand on the pedals during the first seconds of the tests, and vigorous verbal encouragement was given throughout. An active recovery period of three min followed each test. Participants maintained their normal intake of food and fluids, but they abstained from physical exercise and consumption of alcohol and caffeine for 1 day, and ate no food for two hours before testing.

Phase I examined the respective reliability of WAnT_20_ and WAnT_30_ protocols. Forty one athletes performed each test twice, on separate days, at the same time of day, and in a randomized order. PPO (highest 5-s output), PO_min_ (lowest 5-s PO), MPO (average PO throughout the test), and FI (percentage drop in power output from PPO to PO_min_) were determined for each of the two protocols [4].

In phase II, 31 athletes performed a single WAnT_30_. The standard WAnT_30_ indices (as detailed above) were used as criterion indices, while the corresponding data from the first 20 s of the same test were used as predictors.

In phase III, 31 athletes performed both WAnT_30_ (criterion) and WAnT_20_ (predictor) tests. As a result, the agreement between the measured indices from the WAnT_30_ and predicted indices developed from phase II was quantified, using the 95% limits of agreement method (LoA).

### Statistical Analyses

Data analyses were performed using SPSS software (version 19.0 for Windows) and MedCalc version 11.1.1.0. Means and standard deviations (SD) were calculated for each variable. The normality of appropriate data sets was confirmed by applying the Anderson-Darling test of normality [1], allowing hypotheses to be tested by parametric statistical techniques. A maximum a priori α of 0.05 was applied throughout.

In phase I, we evaluated the hypothesis that the sample means of test and retest values did not differ, using a paired sample *t*-test. To help protect against type II errors, an estimate of the effect size (dz) was made to determine if differences between trials were trivial [21]. Reliability was assessed by calculating intra-class correlation coefficients (ICC) model 3,1 [22]. ICCs>0.90 were considered as high, 0.80 to 0.90 as moderate, and <0.80 as low. The absolute reliability of WAnT_20_ and WAnT_30_ values was expressed as the standard error of measurements (SEM) [23]. To complement the SEM, the smallest worthwhile change (SWC) was determined by rearrangement of Cohen's *d* effect size calculation, where the smallest worthwhile effect (0.2) is multiplied by the between-subject SD [24]. By comparing SWC with SEM, test sensitivity was determined, using the thresholds proposed by Lexell and Downham [24]. When SEM was ≤ SWC, the test's capacity to detect change was considered “good”, when SEM was equal to SWC it was considered “satisfactory”, and when SEM was ≥ SWC the test was rated as “marginal”. The square root of the mean square error (MSE) was used to calculate SEM (SEM  =  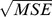) [10], [25]. The SEM% (SEM/mean×100 of all measurements from both sessions) was also calculated in order to compare the WAnT_20_ and WAnT_30_ indices. Before reporting the relevant data in the units of measurement, heteroscedasticity was assessed. Since heteroscedasticity was found in the present data, a log transformation was applied, an antilog (back transformation) was performed to give values that could be interpreted in relation to the original scale [26].

In phase II, a stepwise regression equation was developed to predict MPO, PO_min_, and FI from data collected during the first 20 s of the WAnT_30_. In phase III, Bland-Altman plots were used to determine the goodness of fit for the developed prediction equations [27].

## Results

Summary results for WAnT_20_ and WAnT_30_ tests and retests are shown in Tables 1 and 2. Residual data for WAnT_20_ and WAnT_30_ test and retest were normally distributed (Anderson-Darling p = 0.13–0.8), with no significant differences between test and retest outcomes for WAnT_20_ or WAnT_30_. The relative and absolute reliability of PO_min_ and FI were poor for both test durations; the SEMs for these two measures were larger than their respective SWCs, indicating that both were of marginal value (Table 1 and 2). However, the PPO and MPO values satisfied the ICC_(3,1)_ criterion of high relative reliability, and this was confirmed by SWCs values that were larger than their SEMs counterparts (Table 1 and 2).

**Table 1:** Reliability of the WAnT_30_ (values are means ±SD).

Variable	Trial one	Trial two	p	dz	Heter Coeff	ICC	SWC	SEM
PPO (Logarithms)	94±0.04§ (991±151 W)	2.95±0.05§ (989±145 W)	0.41	0.13	r = −0.40 (p = 0.06)	0.95	0.1	0.01
MPO (W)	595±61§	592±64§	0.73	0.04	r = 0.08 (p = 0.70)	0.90	27.54	12.95
PO_min_ (W)	343±56§	370±58§	0.06	0.42	r = −0.15 (p = 0.46)	0.57	13.46	48.38
FI (%)	56.0±4.16§	56.8±5.4§	0.28	0.19	r = 0.02 (p = 0.95)	0.76	1.09	9.50

PPO: Peak Power Output; MPO: Mean Power Output; PO_min_: Minimal Power Output;FI: Fatigue Index;

§ :Accept normality; dz: Cohen'd; Heter Coeff: Heteroscedasticity coefficient; ICC: Intra Class Correlation Coefficient; SWC: Smollest Worth While Change; SEM: Standard Error of Measurement.

**Table 2:** Reliability of WAnT_20_ (values are means ±SD).

Variable	Trial one	Trial two	p	dz	Hetero Coeff	ICC	SWC	SEM
PPO (W)	991±146§	999±153§	0.26	0.22	r = −0.30; p = 0.13	0.98	30.64	25.47
MPO (W)	696±113§	703±119§	0.19	0.25	r = −0.18; p = 0.19	0.98	23.88	19.46
POmin (W)	488±101§	496±101§	0.34	0.1	r = −0.11; p = 0.59	0.71	20.28	57.69
FI (%)	49.6±7.9§	50.4±6.6§	0.75	0.1	r = −0.34; p = 0.83	0.53	1.32	5.81

***PPO***: Peak Power Output; ***MPO***: Mean Power Output; ***PO_min_***: Minimal Power Output;***FI***: Fatigue Index;

§ :Accept normality; ***dz***: Cohen'd; ***Heter Coeff***: Heteroscedasticity coefficient; ***ICC***: Intra Class Correlation Coefficient; ***SWC***: Smallest Worth While Change; ***SEM***: Standard Error of Measurement.

Table 3 presents stepwise regression data. The MPO accounted for the greatest coefficient of determination (R^2^ = 0.98).

**Table 3:** Regression equations to estimate WAnT_30_ indices from first 20-s values of the same test.

Variable		Parameter estimate	t	p	Co-linearity index
**MPO_WAnT30_** (R^2^ = 0.98)	Intercept	20.9	1.09		
	PPO	0.24	8.73	0.000	0.51
	P_15_	0.35	5.36	0.000	0.16
	P_20_	0.32	4.67	0.000	0.16
**PO_min WAnT30_** (R^2^ = 0.71)	Intercept	−94.7	−1.62		
	P_5_	0.23	2.24	0.000	0.48
	P_15_	0.46	4.25	0.000	0.48
**IF_WAnT30_** (R^2^ = 0.41)	Intercept	68.0	10.85		
	PPO	0.02	2.97	0.000	0.54
	P_15_	−0.05	−4.67	0.000	0.54

PPO: Peak Power Output; MPO_WAnT30_: Mean Power Output of the WAnT_30_; PO_min WAnT30_: Minimal Power Output of the WAnT_30_; FI_WAnT30_: Fatigue Index of the WAnT_30_; P_5_: Power at the 5^th^ s; P_15_: power at the 15^th^ s; P_20_: Power at the 20^th^ s.

Table 4 indicates that residual errors between measured and estimated MPO, PO_min_, and FI were normally distributed, and that the mean biases were not statistically significant. The raw MPO data showed evidence of heteroscedasticity, with positive coefficients (r = 0.24; p = 0.20). Data were therefore transformed into natural logarithms. The dependent *t*-test performed between the mean log transformed indices for measured and predicted MPOs showed no significant systematic biases (p = 0.08; dz = 0.12). Residual errors were normally distributed, and the 95% ratio-limits of agreement were −0.004±0.017.

**Table 4:** Measured and estimated WAnT_30_ scores (values are means ±SD).

Variable	Measured	Estimated	p	dz	Heter Coeff	LoA	Ratio LoA
MPO (W)	612.3±97.1§	620.5±103.5§	0.08	0.12	r = 0.24 (p = 0.20)	------	0.99×/÷1.04
POmin (W)	359.3±81.1§	352.8±76.7§	0.45	0.13	r = −0.01 (p = 0.96)	6.5±92.7(W)	------
FI (%)	60.7±6.0§	62.4±4.4§	0.08	0.3	r = −0.11 (p = 0.55)	1.6±9.8(%)	------

MPO: Mean Power Output; POmin: Minimal Power Output; FI: Fatigue Index; Heter Coeff: Heteroscedasticity coefficient;

§ :Accept normality; dz: Cohen'd; LoA: Limits of agreement.

## Discussion

The purpose of the present study was to determine the relative and absolute reliability as well as the validity of WAnT_20_ when compared to the standard WAnT_30_. Our results demonstrated that the WAnT_20_ is a reliable tool for the evaluation of the anaerobic performance of the legs in male team sport athletes. Furthermore, it appears that if desired, the traditional WAnT_30_ indices can be predicted accurately, using data collected during the WAnT_20_.

### Relative and Absolute Reliabilities

In this study we analysed the respective test-retest reliabilities of the WAnT_20_ and WAnT_30_ by complementary indices of relative and absolute reliability. Relative reliability is indicated by the ICC [22]. The ICCs for PPO and MPO were very high for both protocols, although tending to be slightly higher for 20 s than for 30 s tests. In contrast, the ICCs for FI and PO_min_ fell below the minimal standard of acceptability for reliability (Tables 1 and 2). The ICC cannot be used as the sole statistical measure of reliability, since it is affected by sample heterogeneity [27]. Consequently, we determined the SEM as a measure of absolute reliability [12]. Retest reliability and measurement errors were comparable between the two test protocols. However, the FI and PO_min_ for both protocols also showed larger coefficients of variation than the MPO and PPO. We conclude that with either protocol, the most sensitive measures for evaluating real change are the MPO and PPO, and that the reliability of the 20 s test is at least as good as the traditional 30 s protocol for these two indices.

We also examined the likelihood that the true values of estimated differences in test outcomes would be substantial (i.e., larger than the SWC). Inspection of Tables 1 and 2 shows that the SWC for PPO and MPO for both test protocols were greater than their SEMs, indicating that these indices have a good ability to detect real changes in anaerobic performance of the legs in team athletes. In contrast, the data for FI and PO_min_ had SWCs much greater than their SEMs, calling into question their use in assessing the anaerobic performance of team athletes. Oliver [25] has suggested that the mathematical procedures involved in calculating fatigue levels can influence their reliability.

### Prediction of the Want_30_ Indices from Data Collected during the First 20 S of the Test

The results from the second phase of our study show that the traditional WAnT_30_ indices can, if desired, be predicted accurately based on data collected during the first 20 seconds of the same test (Table 3). In our study, all participants achieved PPO within the first 5–10 s of the WAnT_30_. It seems that if PPO is the only variable of interest, then the WAnT_30_ could easily be shortened to 10 seconds. However, in order for MPO (R^2^ = 0.97) and PO_min_ (R^2^ = 0.71) to be predicted effectively, the test must continue for 20 seconds. These results agree with the observations of Stickley et al [28] and Laurent et al [10], who found that MPO and PO_min_ could be predicted from the first 20 seconds of a WAnT_30_ in female and male college students, respectively. Unlike these two studies, the present study showed a limited ability to predict FI (R^2^ = 0.41), bringing into question the value of the FI as a measure of relative performance decline during all-out effort.

The development of an anaerobic protocol even shorter than 20 seconds would further reduce detrimental side effects. However, regression equations based on only the first 15 seconds of the WAnT_30_ were less effective in predicting MPO (R^2^ = 0.945), PO_min_ (R^2^ = 0.677), and FI (R^2^ = 0.345). Stickley et al [28] had similar findings in female college students, with coefficients of determination for MPO, PO_min_, and FI of 0.965, 0.796 and 0.548, respectively based upon 15 s tests.

### Validity of the Want_20_ versus the Want_30_

In Phase III of this study, participants tended to a slightly greater PPO during WAnT20 (924±165 W) than during WAnT30 (916±134 W), although this difference was not statistically significant. Significantly greater values for MPO, PO_min_ and FI for WAnT20 (675±118 W, 484±100 W, and 49.7±7.1%, respectively) compared to WAnT30 (612±94 W, 364±80 W, and 60.3±6.5%, respectively) suggest that subject performance fell dramatically in the final 10 seconds of the test. Nevertheless, our results agree with those reported by Laurent et al. [10] and Stickley et al [28], who found that these indices could be predicted by a WAnT20 protocol in both female and male collegiate students. Hachana et al [23] were also able to produce simple regression equations to estimate WAnT30 from a WAnT15 test.

The prediction algorithms developed in these earlier reports were not validated by subsequent independent studies [29]. However, the accuracy of our prediction equations (Table 3) were evaluated by the Bland and Altman method [27]; PO_min_ and FI data were homoscedastic (Table 4), i.e., the calculated limits of agreement remain constant throughout the range of measurement and can therefore be accepted [27]. Indeed, the differences between measured and predicted PO_min_ and FI for male physical education students would be expected to lie within the limits of 6.5±92.8 (W) and 1.6±9.8 (%), respectively. Heteroscedasticity occurs when the random error in data increases as the measured values increase [30]; in such circumstances, it is necessary to transform the original test data into natural logarithms and then repeat the limits of agreement tests [27]. Nevill and Atkinson [31] suggested that if the coefficient of correlation between absolute residual errors and the individual means was positive, but not necessarily statistically significant, it was also desirable to transform raw data into natural logarithms and recalculate the limits of agreement in order to reduce heteroscedasticity. Our MPO raw data showed some evidence of heteroscedasticity (Table 4), and accordingly we transformed these data into natural logarithms for analysis. Recalculating antilogs resulted in a limits of agreement of −0.004±0.017, a mean bias on the ratio scale of 0.99 (exponential −0.004) and an agreement of ±1.04 (exponential 0.017); thus, 95% of the ratios for the sample (log transformed test score divided by log transformed retest score) should lie between the values of 0.95 (0.99÷1.04) and 1.03 (0.99×1.04). Assuming the bias for estimating MPO of 0.004 to be negligible, the predicted and measured WAnT_30_ MPO would differ due to measurement error by no more than 1.7%. To put this limit of agreement into a practical context, if a subject presented with a WAnT_20_ predicted MPO of 500 W, the worst case scenario is that this athlete had an MPO as low as 500×0.95 = 475 W or as high as 500×1.03 = 515 W.

### Limitations

Our findings have been limited to a group of young men engaged in team sports. Further data are needed to confirm that a 20 s protocol is appropriate for assessing the anaerobic performance of those engaged in other types of sport, at other levels of training, in different age groups, and particularly in female participants. The effects of habituation and test learning on a 20 s test are also an important area for future enquiry, as are more quantitative evaluations of the extent of symptoms with 20 and 30 s tests.

## Conclusion

In summary, our study demonstrates that the WAnT_20_ is a reliable tool for measuring the anaerobic performance of the leg muscles in young men trained for team sports, and if desired the data can be used to predict traditional Wingate test indices. In contrast to previous studies, we have shown that WAnT_20_ is reliable and can be used to accurately predict WAnT_30_ indices. Therefore, the WAnT_20_ data can be used by coaches, clinicians, and athletes as reliable and good predictors of the standard WAnT_30_ parameters.

## References

[1] Andersen KL, Shephard RJ, Denolin H, Varnauskas E, Masironi R (1971) Fundamentals of exerice testing. World Health Organisation. Geneva, Switzerland.

[2] Shephard RJ (1982) Physiology and biochemistry of exercise. New York, NY.

[3] Serresse O, Lortie G, Bouchard C, Boulay MR (1988) Estimation of the contribution of the various energy systems during maximal work of short duration. Int J Sports Med 9:456–460. 3253239 10.1055/s-2007-1025051

[4] Bar-Or O (1987) The Wingate anaerobic test. An update on methodology, reliability and validity. Sports Med 4:381–394. 3324256 10.2165/00007256-198704060-00001

[5] Bar-Or O, Dotan R, Inbar O (1977) A 30 s all-out ergometric test: its reliability and validity for anaerobic capacity. Isr J Med Sci 13:126.68022

[6] Hopkins WG (2000) Measures of reliability in sports medicine and science. Sports Med 30:1–15. 10907753 10.2165/00007256-200030010-00001

[7] Zagatto AM, Beck WR, Gobatto CA (2009) Validity of the running anaerobic sprint test for assessing anaerobic power and predicting short-distance performances. J Strength Cond Res 23:1820–1827. 19675478 10.1519/JSC.0b013e3181b3df32

[8] Dal Pupo J, Gheller RG, Dias JA, Rodacki AL, Moro AR, et al (2013) Reliability and validity of the 30-s continuous jump test for anaerobic fitness evaluation. J Sci Med Sport 17:650–655. 24606832 10.1016/j.jsams.2013.09.007

[9] Ozkaya O, Colakoglu M, Kuzucu EO, Delextrat A (2014) An elliptical trainer may render the Wingate all-out test more anaerobic. J Strength Cond Res 28:643–650. 23924890 10.1519/JSC.0b013e3182a20f77

[10] Laurent CMJ, Meyers MC, Robinson CA, Green JM (2007) Cross-validation of the 20- versus 30-s Wingate anaerobic test. Eur J Appl Physiol 100:645–651. 17429677 10.1007/s00421-007-0454-3

[11] Allen DG, Westerblad H, Lee JA, Lannergren J (1992) Role of excitation-contraction coupling in muscle fatigue. Sports Med 13:116–126. 1313991 10.2165/00007256-199213020-00007

[12] Vincent S, Berthon P, Zouhal H, Moussa E, Catheline M, et al (2004) Plasma glucose, insulin and catecholamine responses to a Wingate test in physically active women and men. Eur J Appl Physiol 91:15–21. 14551777 10.1007/s00421-003-0957-5

[13] Jacobs I, Bar-Or O, Karlsson J, Dotan R, Tesch P, et al (1982) Changes in muscle metabolites in females with 30-s exhaustive exercise. Med Sci Sports Exerc 14:457–460. 7162392 10.1249/00005768-198206000-00009

[14] Jacobs PL, Mahoney ET, Johnson B (2003) Reliability of arm Wingate Anaerobic Testing in persons with complete paraplegia. J Spinal Cord Med 26:141–144. 12828291 10.1080/10790268.2003.11753674

[15] Smith JC, Hill DW (1991) Contribution of energy systems during a Wingate power test. Br J Sports Med 25:196–199. 1839780 10.1136/bjsm.25.4.196 PMC1479034

[16] Kavanagh MF, Jacobs I (1988) Breath-by-breath oxygen consumption during performance of the Wingate Test. Can J Sport Sci 13:91–93. 3359368

[17] Medbo JI, Tabata I (1989) Relative importance of aerobic and anaerobic energy release during short-lasting exhausting bicycle exercise. J Appl Physiol (1985) 67:1881–1886. 2600022 10.1152/jappl.1989.67.5.1881

[18] Stevens GHJ, Wilson BW, Raven PB (1986) Aerobic contribution to the Wingate test. Med Sci Sports Exerc 18 (Suppl).

[19] Graham JE, Boatwright JD, Hunskor MJ, Howell DC (2003) Effect of active vs. Passive recovery on repeat suicide run time. J Strength Cond Res: 338–341. 10.1519/1533-4287(2003)017<0338:eoavpr>2.0.co;2 12741874

[20] Vanderford ML, Meyers MC, Skelly WA, Stewart CC, Hamilton KL (2004) Physiological and sport-specific skill response of olympic youth soccer athletes. J Strength Cond Res 18:334–342. 15142015 10.1519/R-11922.1

[21] Faul F, Erdfelder E, Lang AG, Buchner A (2007) G*Power 3: a flexible statistical power analysis program for the social, behavioral, and biomedical sciences. Behav Res Methods 39:175–191. 17695343 10.3758/bf03193146

[22] Wang CY, Sheu CF, Protas EJ (2009) Test-retest reliability and measurement errors of six mobility tests in the community-dwelling elderly. Asian J Gerontol Geriatr 4:8–13.

[23] Hachana Y, Attia A, Nassib S, Shephard RJ, Chelly MS (2012) Test-retest reliability, criterion-related validity, and minimal detectable change of score on an abbreviated Wingate test for field sport participants. J Strength Cond Res 26:1324–1330. 22516906 10.1519/JSC.0b013e3182305485

[24] Lexell JE, Downham DY (2005) How to assess the reliability of measurements in rehabilitation. Am J Phys Med Rehabil 84:719–723. 16141752 10.1097/01.phm.0000176452.17771.20

[25] Oliver JL (2009) Is a fatigue index a worthwhile measure of repeated sprint ability? J Sci Med Sport 12:20–23. 18078786 10.1016/j.jsams.2007.10.010

[26] Nevill A (1996) Validity and measurement agreement in sports performance. J Sports Sci 14:199. 8809712 10.1080/02640419608727704

[27] Bland JM, Altman DA (1986) Statistical methods for assessing agreement between two methods of clinical measurement. Lancet 327(8476):307–310. 2868172

[28] Stickley CD, Hetzler RK, Kimura IF (2008) Prediction of anaerobic power values from an abbreviated WAnT protocol. J Strength Cond Res 22:958–965. 18438215 10.1519/JSC.0b013e31816a906e

[29] Cooper SM, Baker JS, Eaton ZE, Matthews N (2004) A simple multistage field test for the prediction of anaerobic capacity in female games players. Br J Sports Med 38:784–789. 15562181 10.1136/bjsm.2004.012229 PMC1724973

[30] Atkinson G, Nevill AM (1998) Statistical methods for assessing measurement error (reliability) in variables relevant to sports medicine. Sports Med 26:217–238. 9820922 10.2165/00007256-199826040-00002

[31] Nevill AM, Atkinson G (1997) Assessing agreement between measurements recorded on a ratio scale in sports medicine and sports science. Br J Sports Med 31:314–318. 9429009 10.1136/bjsm.31.4.314 PMC1332566

